# Phototrophic Fe(II)‐Oxidizers Can Use Multiple (In)Organic Substrates Simultaneously

**DOI:** 10.1111/1758-2229.70428

**Published:** 2026-11-04

**Authors:** Verena Nikeleit, Giorgio Bianchini, Jimena C. Lopez‐Rivoldi, Marie Mergenthaler, Josia Reinhold, Patricia Sanchez‐Baracaldo, James M. Byrne, Andreas Kappler, Casey Bryce

**Affiliations:** ^1^ Department of Geoscience University of Tübingen Tübingen Germany; ^2^ NORCE Norwegian Research AS Bergen Norway; ^3^ School of Geographical Sciences University of Bristol Bristol UK; ^4^ Department of Life Sciences University of Bath Bath UK; ^5^ School of Earth Sciences University of Bristol Bristol UK; ^6^ Cluster of Excellence: EXC 2124: Controlling Microbes to Fight Infections Tübingen Germany

**Keywords:** Fe(II) oxidation, metabolic flexibility, photoferrotroph

## Abstract

Anoxygenic phototrophs are metabolically flexible bacteria. However, it is unclear how Fe(II) oxidation is affected by the simultaneous availability of organic or inorganic substrates. We therefore cultivated six green‐ and purple non‐sulfur photoferrotrophs from freshwater and marine environments with 2 mM Fe(II), amended with 0.6 mM organic co‐substrate or H_2_. We found that photoferrotrophs generally utilized organics and Fe(II) simultaneously, and that two marine strains preferred Fe(II) over H_2_. For some photoferrotrophs, Fe(II) oxidation rates were significantly enhanced by acetate, lactate, and glucose, whereas H_2_ typically slowed Fe(II) oxidation. All phototrophic Fe(II)‐oxidizers oxidized 80%–100% of the Fe(II) by the end of the experiment except for *R. robiginosum* , where Fe(II) oxidation reached only 52%–73%. We further observed for the first time that *R. palustris* TIE‐1 can grow with butyrate and glucose and that *R. iodosum* can utilize glucose. Comparison of genomic predictions with experimentally observed substrate consumption revealed discrepancies in four strains. Overall, our results demonstrate that Fe(II) remains an important electron donor despite the availability of alternative substrates, suggesting that maintaining photoferrotrophy is a common trait of photoferrotrophs and likely contributes to iron cycling in natural environments.

## Graphical abstract

We demonstrated that photoferrotrophs generally utilized organics and Fe(II) simultaneously, and that two of the strains prefer Fe(II) over H_2_. For some photoferrotrophs, Fe(II) oxidation rates were significantly enhanced by the presence of acetate, lactate, and glucose. On the other hand, H_2_ typically slowed down Fe(II) oxidation.

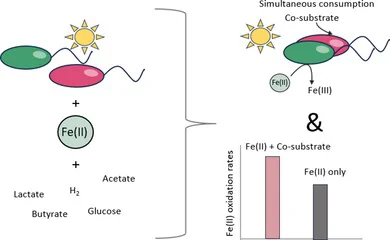

## 1 Introduction

Anoxygenic phototrophs are widespread and play a crucial role in many environments, such as stratified lakes, freshwater and marine sediments where they contribute to energy flow and nutrient cycling (Bryce, Blackwell, et al. 2018; Crowe et al. 2017; Laufer et al. 2017; Otte et al. 2018; Walter et al. 2014). Anoxygenic phototrophs have been historically classified into green sulfur and purple sulfur or non‐sulfur bacteria based on the morphological studies of Winogradsky that described the bacteriochlorophyll composition and ability to use and store sulfur (Winogradsky 1888). Anoxygenic phototrophs use light to gain energy but, in comparison to oxygenic phototrophs, no oxygen is produced. Instead of using water as an electron donor as in oxygenic photosynthesis, anoxygenic phototrophs use alternative substrates such as organic and inorganic compounds. By using organic substrates like acetate, lactate, glucose, and butyrate they grow photoheterotrophically and can use the organics as an electron donor as well as a carbon source for biomass. However, when grown photoautotrophically, inorganic substrates like H_2_, H_2_S, S_2_O_3_^2−^ and Fe(II) are used as electron donors to fix CO_2_ into biomass. In general, green sulfur bacteria are primarily adapted to the use of inorganic electron donors coupled to autotrophic CO_2_ fixation, whereas purple phototrophs exhibit greater metabolic flexibility and utilize a broad range of organic substrates during photoheterotrophic and photomixotrophic growth (Imhoff 1995; Madigan and Jung 2009). Many anoxygenic phototrophs are also capable of photomixotrophic growth, whereby organic and inorganic substrates can be consumed simultaneously or sequentially. Under these conditions, organic compounds may serve as carbon and/or electron donors while inorganic electron donors provide additional electrons and reducing equivalents for metabolism. This wide range of possible substrates and the ability to use multiple substrates make anoxygenic phototrophs ubiquitous as they can use, and switch between, a vast variety of substrates.

One group of anoxygenic phototrophs that has gained particular attention are photoferrotrophs, which were only discovered 33 years ago but have been intensively studied, particularly due to their suggested importance for primary productivity in the Earth's ancient ferruginous oceans (Ehrenreich and Widdel 1994; Kappler et al. 2005; Thompson et al. 2019; Widdel et al. 1993). These phototrophs are able to use Fe(II) as an electron donor for photosynthesis, thereby linking iron cycling to carbon fixation. Iron is a ubiquitous redox‐active element that is linked to other crucial element cycles through both metabolism and the formation and dissolution of Fe minerals, which affects the bioavailability and accessibility of other toxins, pollutants, and nutrients (Eickhoff et al. 2014; Kappler et al. 2021; Mu et al. 2016; Tipping 1981). To date, only a few isolates have been obtained, which originated from various environments. From freshwater environments, *Chlorobium ferrooxidans* KoFox, *Rhodobacter ferrooxidans* SW2, and *Rhodopseudomonas palustris* TIE‐1 have been isolated (Ehrenreich and Widdel 1994; Heising et al. 1999; Jiao et al. 2005). From marine environments, *Chlorobium* sp. N1, *Rhodovulum iodosum* , and *Rhodovulum robiginosum* are used as model strains (Laufer et al. 2017; Straub et al. 1999). Because many photoferrotrophs are capable of photomixotrophic growth, it remains unclear whether Fe(II) is utilized alongside alternative electron donors or only after their depletion. Determining these substrate preferences is essential for assessing the environmental significance of photoferrotrophy in the presence of competing electron donors.

This question is particularly relevant because photoferrotrophs are rarely exposed to Fe(II) as the sole electron donor in nature. Ferruginous environments generally contain mixtures of organic and inorganic substrates, including H_2_ and dissolved organic compounds. Under such conditions, it could be that photoferrotrophs preferentially utilize these alternative substrates rather than Fe(II). If so, the contribution of photoferrotrophs to environmental iron cycling could be substantially lower than currently assumed. In contrast, if Fe(II) oxidation continues or is even stimulated in the presence of alternative substrates, photoferrotrophy may remain an important driver of iron cycling. This question is important because Fe(II) oxidation influences not only microbial growth but also Fe(III) mineral formation, nutrient availability, trace metal cycling, and carbon cycling.

The few studies that have investigated the effect of alternative substrates on photoferrotrophs found that substrate utilization could be strain specific. For example, *R. ferrooxidans* SW2 sequentially consumed organics (acetate or glucose) before oxidizing Fe(II), whereas *Chromatium* L7, a purple‐sulfur bacterium, simultaneously consumed organics (acetate or glucose) and Fe(II) (Ehrenreich and Widdel 1994). The identity of the organic compound also appears to be important for determining how organic and inorganic substrates are used. For example, in *R. palustris* TIE‐1, simultaneous consumption was observed for Fe(II) and glucose or lactate, but sequential consumption was observed for acetate, pyruvate, or butyrate over Fe(II). In the same study, they observed that co‐substrate utilization appeared to enhance Fe(II) oxidation rates (Melton et al. 2014; Nikeleit, Maisch, et al. 2024). For inorganic substrates, Croal and colleagues showed that Fe(II) oxidation rates for the photoferrotrophs *R. ferrooxidans* SW2 and *R. palustris* TIE‐1 decreased in the presence of H_2_ (Croal et al. 2009).

While these studies have provided important insights, they were typically restricted to one or two strains, investigated different combinations of substrates, used different Fe(II) sources, Fe(II) concentrations, co‐substrate concentrations, and light conditions. As a result, direct comparisons among strains are difficult, and it remains unclear whether the persistence or stimulation of Fe(II) oxidation in the presence of alternative substrates represents a general feature of photoferrotrophs or merely the behaviour of a few well‐studied model organisms. To address this knowledge gap, we performed a systematic comparison of six photoferrotrophic model strains representing both green sulfur and purple phototrophs and from both freshwater and marine environments. In contrast to previous studies, all strains were cultivated using the same Fe(II) source, environmentally relevant substrate concentrations, and light conditions. We investigated the interactions between Fe(II) and four organic substrates (acetate, lactate, glucose, and butyrate) as well as the inorganic substrate H_2_. By studying a diverse set of photoferrotrophs under identical conditions, we want to assess whether Fe(II) oxidation is maintained or enhanced in the presence of alternative electron donors like in previous publications, whether this behaviour is shared across photoferrotrophs and ultimately whether photoferrotrophy remains an important contributor to iron cycling in environments where multiple electron donors coexist.

## 2 Material and Methods

### 2.1 Substrate Preference Experiments With Fe(II) and Different Organics

Five different photoferrotrophic model strains were chosen: *R. ferrooxidans* SW2, *R. iodosum, R. robiginosum, C. ferrooxidans* KoFox, *and Chlorobium* sp. N1. Results for *R. palustris* TIE‐1 have been published previously in Nikeleit, Maisch, et al. 2024. All strains used in this study are maintained in the Geomicrobiology Group of Prof. Kappler in Tuebingen, Germany. The phototrophic bacteria were grown for two transfers on H_2_/CO_2_ (80:20; v:v) prior to the experiment to avoid bias towards any of the experimental substrates, except for *R. iodosum* , where a pre‐culture grown on FeCl_2_ was used due to time limitations. 50 mL media was added to 118 mL serum bottles and inoculated with 2% of the pre‐grown cultures. Each bacterium was cultivated with a specific medium composition that can be found in (Table S1). For media preparation, the medium specific salts were dissolved in 1 L ultrapure water and autoclaved at 121°C for 20 min in a Widdel flask. After autoclaving, the media were cooled to room temperature under an N_2_/CO_2_ (90:10) atmosphere and buffered with anoxic 22 mM bicarbonate buffer. Aliquots of 1 mL/L sterile filtered 7‐vitamin solution (Widdel and Pfenning 1981), trace element solution (Widdel 1983), and selenite‐tungstate solution (Widdel and Bak 1992) were added and the pH was adjusted to 7 with anoxic and sterile 0.5 M Na_2_CO_3_ or 1 M HCl. In the first setup, only 2 mM FeCl_2_ was added as a control. In the second setup, FeCl_2_ and an organic substrate (0.6 mM of either acetate (Ac), lactate (Lc), butyrate (Bu), or glucose (Glu)) were added. Biological setups were run in triplicates and abiotic controls (same setup without the addition of bacterial cultures) were performed in duplicates. The bottles were placed randomly in a light incubator at 20°C with 25 ± 7 μmol photons/s/m^2^. Samples were taken in a glovebox with 100% N_2_ atmosphere for quantification of Fe, organic, and cell numbers.

### 2.2 Substrate Preference Test With Fe(II) and H_2_

For this experiment, we used the five photoferrotrophs listed above plus *R. palustris* TIE‐1, which were grown for two transfers on acetate or lactate (except for *Chlorobium* sp. N1 which was pre‐grown on FeCl_2_ which struggled to grow on acetate or lactate). A sacrificial setup with three biological bottles and one abiotic control was built using Hungate tubes. 9.5 mL media containing 2 mM Fe(II) were added to sterile Hungate tubes and 0.5 mL inoculum was added. The headspace was exchanged with H_2_/CO_2_ (80:20 v:v). The bottles were placed randomly in a light incubator at 20°C with 25 ± 7 μmol photons/s/m^2^. Gas samples were taken sterilely at the lab bench and samples for Fe and cells were taken in a glovebox with a 100% N_2_ atmosphere.

### 2.3 Fe Quantification

Fe(II) and total Fe were quantified spectrophotometrically with the Ferrozine assay modified after (Hegler et al. 2008). 0.1 mL samples were diluted in 0.9 mL of 1 M HCl and stored at 4°C. The ferrozine‐Fe(II) complex was quantified at 562 nm using a microtiter plate reader (Thermo Scientific Multiscan, Thermo Fisher Scientific). Ferrozine measurements were conducted in triplicates.

### 2.4 H_2_ Quantification

3 mL gas was taken and transferred into a N_2_ purged headspace vial. Gas samples were analysed on a TraceGC1300 (ThermoFisher Scientific, modified by S + HA analytics, Germany), with a 30 m long, 0.53 mm ID Molsieve column connected to a Pulse Discharge Detector.

### 2.5 Short‐Chain Fatty Acid Quantification

Acetate, lactate, butyrate, and glucose were quantified by HPLC. Samples were centrifuged at 20,000 for 5 min to remove cells and minerals. A Shimadzu Prominence HPLC with a LC‐20AT solvent delivery unit, CTO‐10ASvp column oven, and a RID‐20a refractive index detector was used.

### 2.6 Cell Quantification

Cells were counted using a flow cytometer equipped with a 488 nm laser as an excitation source (Attune Nxt flow cytometer, Thermo Fisher Scientific). 180 μL of samples were fixed with 20 μL formaldehyde solution (CH_2_O). Samples were stored at 5°C until measurement. Minerals were dissolved by adding 200 μL of sterile, anoxic 100 mM Fe(II) ethylenediammonium sulfate tetrahydrate (Fe‐EDAS, FeSO_4_ × C_2_H_8_N_2_H_2_SO_4_ × 4H_2_O) and 600 μL oxalate solution (0.23 M (NH_4_)_2_C_2_O_4_ × H_2_O and 0.17 M C_2_H_2_O_4_, pH 7). After 10 min, BacLight Green stain (Thermo Fisher Scientific, 1 μL stain/1 mL sample) was added and 200 μL of sample was distributed in triplicates in 96‐well plates. The plate was incubated for 15 min in the dark before measuring. Cells were distinguished from noise or debris based on their properties in the side scatter and BL1 channel (with emission filter 530/30 nm). All measurements were conducted in triplicates and the results reported as an average.

### 2.7 Bioinformatics

Genome and annotated proteome sequences for the strains (Table S2) were downloaded from the RefSeq database (O'Leary et al. 2016), with the exception of *R. robiginosum* , for which no sequenced genome was available (Bianchini et al. 2025). The presence of substrate utilization gene markers in the annotated proteomes was assessed using the hmmsearch program from HMMer v3.4 (Eddy 2011), employing HMM profiles (Table S3) downloaded from the NCBI Protein Family Models (Wang et al. 2023) and FeGenie (Garber et al. 2020) databases. For genes where an HMM was not available, blastp v2.12.0+ (Camacho et al. 2009) was used, with query sequences (Table S4) obtained from the UniProt database (Bateman et al. 2021) and from previous studies (Cardona 2018; Lü et al. 2017). Each gene was asserted to be present when a hit was found with a score higher than the GA threshold included in the HMM (Table S3) or than the BLAST score threshold we determined (Table S4); hits close to the thresholds were manually inspected to confirm their exclusion (Tables S5 and S6). To further reduce the likelihood of false negatives, BATH (commit 128e0cb, Krause et al. 2024) and tblastn v2.12.0+ were used to search for genes within the unannotated genome sequences, using the same HMMs and query sequences as above.

### 2.8 Statistical Analysis

Differences between conditions were statistically evaluated. Welsh's *t*‐test was used to account for unequal variances between each sample treatment (3 bottles per treatment). We chose a significance threshold of *α* = 0.05.

## 3 Results

### 3.1 Genetic Potential for Metabolizing Different Inorganic and Organic Substrates

Genomes are available for all investigated strains except *R. robiginosum* (Bianchini et al. 2025). We used this publicly available information to predict the potential different metabolic capabilities of different phototrophic Fe(II)‐oxidizers. While the presence of genes associated with specific pathways suggests metabolic potential it does not necessarily reflect functional expression or activity. When investigating the potential usage of inorganic substrates, we focused on nitrogen and sulfur species, Fe(II) and H_2_ (Table 1). The same gene for Fe(II) oxidation was found in both green sulfur bacteria (*cyc2*), while two purple non‐sulfur bacteria use different mechanisms: *R. ferrooxidans* SW2 uses the *fox* operon, and *R. palustris* TIE‐1 the *pio* operon. An Fe(II) oxidase in *R. iodosum* could not be identified. [NiFe] hydrogenase was found in all phototrophs and *hypA* in all except *Chlorobium* sp. N1. Regarding the potential for nitrogen species usage, all phototrophs have nitrogen fixing genes (*nifD*, *nifH* and *nifK*) whereas only *R. iodosum* shows genetic potential for nitrate reduction (*narG*) (Table 1). *R. ferrooxidans* SW2 and *R. palustris* TIE‐1 have nitrite reduction genes (*nirS and nirK*). Sulfide reductase (*sqr*) is present in the two green sulfur phototrophs and in *R. ferrooxidans* SW2; Chlorobium sp. N1 also has additionally a sulfite reductase gene (*dsrA*). *R. iodosum* and *R. palustris* TIE‐1 have a variety of genes related to using thiosulfate (*soxA*, *soxZ*, *soxY* and *tsdA*), sulfite (*soxC*), sulfate/sulfur (*soxB*, *soxX*) and sulfide (*fccB*, *sqr*).

**TABLE 1:** Metabolic flexibility marker genes. For each available genome (GSB: Green sulfur bacteria; PSB: Purple non‐sulfur bacteria), the figure shows the presence/absence of genes involved in the metabolism of inorganic and organic substrates.

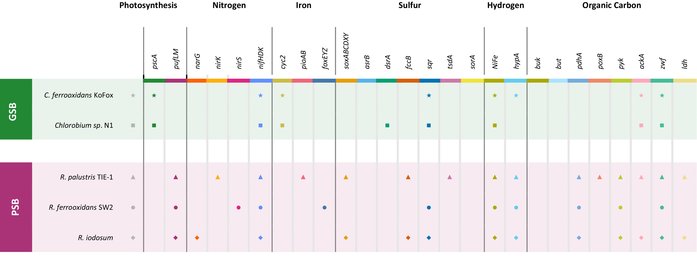

In terms of organic substrate usage, genes for using butyrate (*buk and but*) were not found in any of the phototrophs whereas all had a glucose dehydrogenase gene (*zwf*). *C. ferrooxidans KoFox, Chlorobium* sp. N1 and *R. iodosum* have acetate kinase (*ackA*). Pyruvate dehydrogenase (*pdhA and poxB*) and pyruvate kinase (*pyk*) were only found in the purple non‐sulfur phototrophs. Additionally, lactate dehydrogenase (*ldh*) was only found in *R. iodosum* and *R. palustris* TIE‐1. Overall, the purple non‐sulfur bacteria appear to have the genetic potential for more flexible metabolisms whereas the green sulfur bacteria are more limited, though not restricted to Fe(II) oxidation.

### 3.2 Substrate Preference of Photoferrotrophs Between Fe(II) and Inorganic (H_2_ ) or Organic Substrates

In the substrate preference experiments, all phototrophic Fe(II)‐oxidizers oxidized up to 80%–100% of the Fe(II) at the end of the experiment except for *R. robiginosum* where Fe(II) oxidation only reached 52%–73% (Table 2). However, different substrate preferences (Table 2), Fe(II) oxidation rates (Figure 1) and cell numbers (Figure 2) were observed and are discussed for each individual strain below. We also compared the measured cell numbers to the theoretical cell number using the following assumptions as previously applied by Nikeleit, Maisch, et al. 2024: (i) one cell weighs 1 pg (dry weight consisting of CH_2_O), (ii) for the Fe(II) only setup we assumed all electrons are used for fixing CO_2_ into biomass (CO_2_ ➔ CH_2_O; 4 electrons) and (iii) for organic substances we assumed 90% of the other organic substrates tested in our study were also converted into biomass (McKinlay and Harwood 2010). This calculation predicts that cells grown with Fe(II) only would have 1.2 × 10^7^ cells/mL, 4.4 × 10^7^ cells/mL for Fe(II) plus acetate, 6.0 × 10^7^ cells/mL for Fe(II) plus lactate, 7.7 × 10^7^ cells/mL for Fe(II) plus butyrate and 1.1 × 10^8^ cells/mL for Fe(II) plus glucose.

**TABLE 2:** Schematic summary of substrate preference, Fe(II) oxidation extent, organic (acetate (Ac), lactate (Lc), pyruvate (Py), butyrate (Bu), or glucose (Glu)) and H_2_ consumption extent for *R. palustris* TIE‐1, R. ferrooxidans SW2, *R. iodosum* , *R. robiginosum* , *C. ferrooxidans* KoFox, and *Chlorobium* sp. N1.

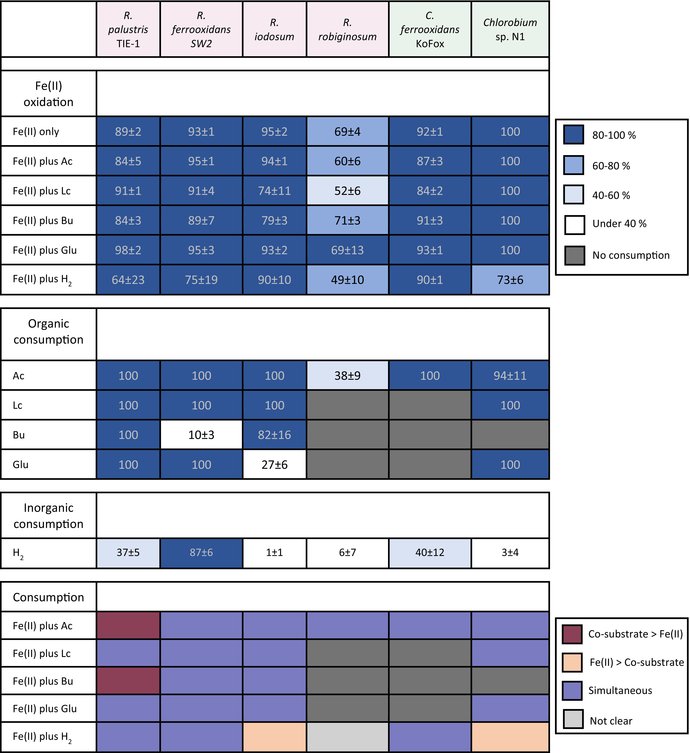

*Note:* Extent of Fe(II) oxidation, organic and inorganic substrate consumption is indicated by a dark blue (high extent) gradient to white (low extent); dark grey indicates no consumption. For different consumption scenarios dark red (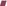) indicates preferred consumption of co‐substrates over Fe(II), beige (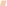) indicates preferred consumption of Fe(II) over co‐substrate, violet (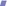) indicates simultaneous consumption and light grey (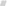) indicates no clear trend.

**FIGURE 1:**
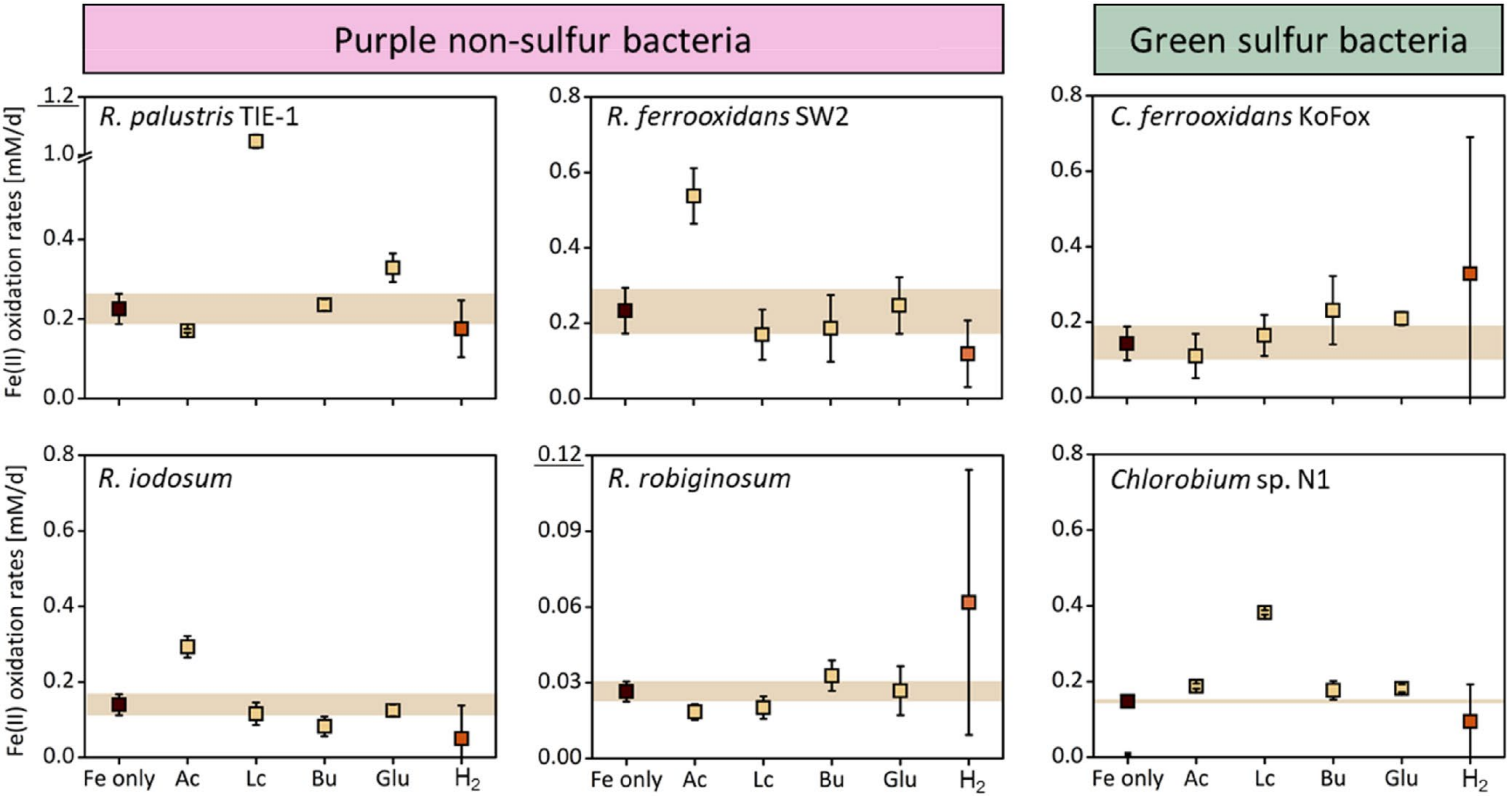
Fe(II) oxidation rates of photoferrotrophs: Panels show the Fe(II) oxidation rates of *R. palustris* TIE‐1, *R. ferrooxidans* SW2, *R. iodosum* , *R. robiginosum* , *C. ferrooxidans* KoFox and *Chlorobium* sp. N1 incubated with Fe(II) only, Fe(II) plus acetate (Ac), Fe(II) plus lactate (Lc), Fe(II) plus butyrate (Bu) or Fe(II) plus glucose (Glu). Values were calculated from triplicates and show standard deviation. The dark brown point shows the Fe(II) oxidation rate when only Fe(II) was added and the light brown background shows the standard deviation of this setup. Underscore values on y‐axis indicates different scale compared to others. Data from *R. palustris* TIE‐1 are taken from (Nikeleit, Maisch, et al. 2024).

**FIGURE 2:**
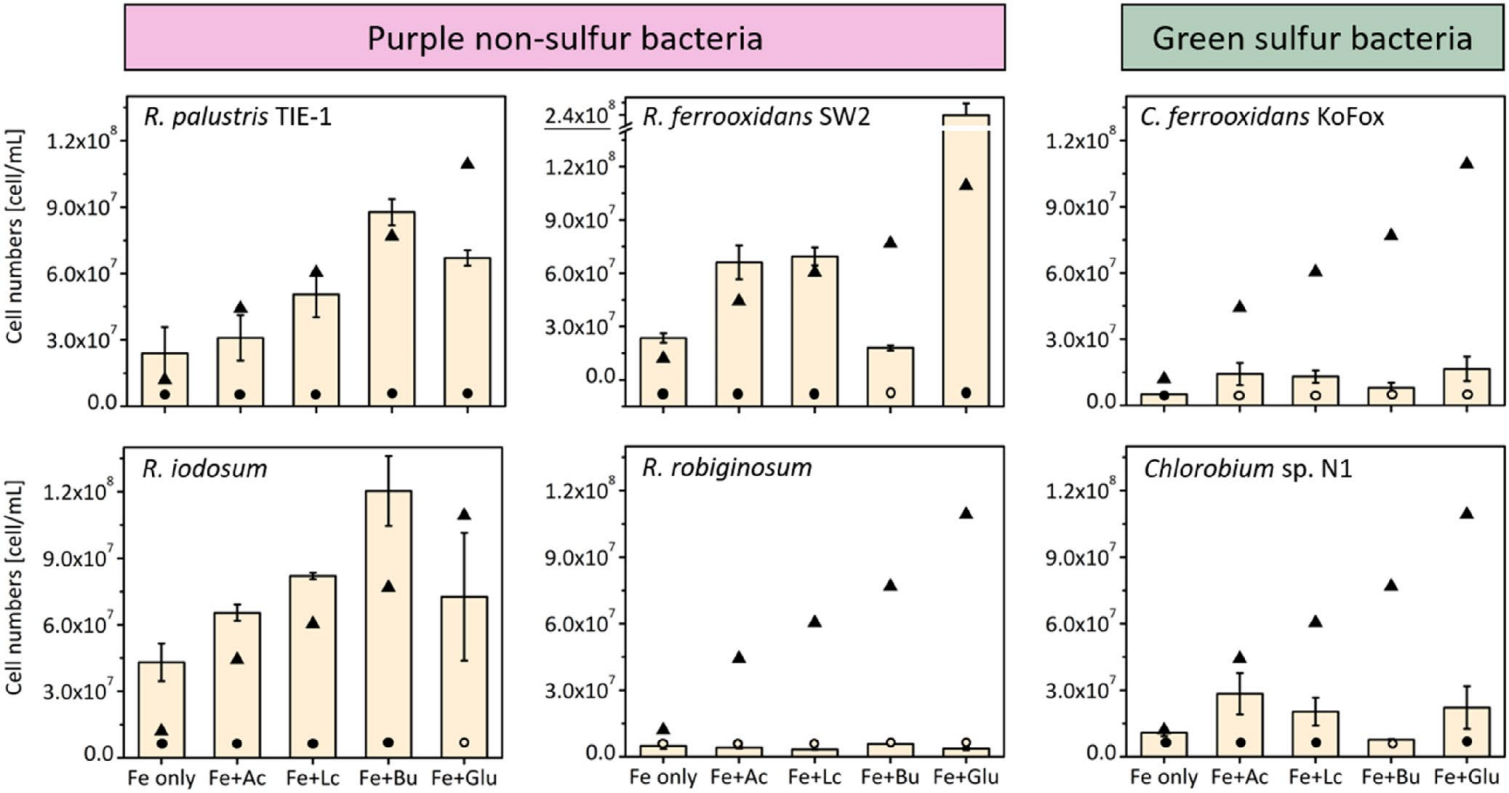
Panels show the cell numbers of *R. palustris* TIE‐1, R. ferrrooxidans SW2, *R. iodosum* , *R. robiginosum* , *C. ferrooxidans KoFox* and *Chlorobium* sp. N1 incubated with Fe(II) only, acetate (Ac), lactate (Lc), butyrate (Bu) or glucose (Glu). Data were measured in triplicates and show standard deviation. Data from *R. palustris* TIE‐1 are from a previous publication (Nikeleit, Maisch, et al. 2024). The black triangle show the theoretical calculated cell numbers. Filled circle indicate that Fe(II) and organics were used completely and not filled circle indicate that at least one substrate was only partial or not consumed.

#### 3.2.1 Substrate Preference of *R. ferrooxidans* SW2

In the substrate preference experiments with different organics and Fe(II), the purple non‐sulfur bacterium *R. ferrooxidans* SW2 was able to use all 4 tested organic substances (acetate, lactate, butyrate and glucose) simultaneously with Fe(II), although only 10% ± 3% of the butyrate was consumed (Table 2, Figure S1). Fe(II) oxidation reached between 89% and 95% completion in all setups. Fe(II) oxidation rates with Fe(II) only were 0.23 ± 0.06 mM/d and no significant changes in Fe(II) oxidation rates were observed with co‐substrates (Figure 1). Only the setup with Fe(II) plus acetate showed a higher Fe(II) oxidation rate with 0.54 ± 0.07 mM/d, which was significantly higher than the rate obtained with Fe(II) only (*p* = 0.005) (Table S7). Cell numbers in the setup with Fe(II) only (2.4 ± 0.3 × 10^7^ cells/mL) and Fe(II) plus butyrate (1.8 ± 0.2 × 10^7^ cells/mL) were similar which was expected given the low amount of butyrate consumed (Figure 2). In setups containing Fe(II) plus acetate (6.6 ± 1.0 × 10^7^ cells/mL) and Fe(II) plus lactate (7.0 ± 0.5 × 10^7^ cells/mL), cell numbers were also similar. The highest cell numbers, double what we would theoretically expect, were found in the setup with Fe(II) plus glucose (2.4 ± 0.4 × 10^8^ cells/mL). In the substrate preference test with Fe(II) and H_2_, simultaneous consumption was observed with no lag phase for both substrates (Table 2, Figure S6). Fe(II) was oxidized with a rate of 0.12 ± 0.09 mM/d to an extent of 75% ± 18% and 87% ± 6% of H_2_ was used up. Both abiotic controls stayed stable over the period of the experiment.

In summary, *R. ferrooxidans* SW2 could use all substrates (butyrate only partially) and consumed them simultaneously with Fe(II). Additionally, Fe(II) oxidation rates significantly increased with Fe(II) plus acetate.

#### 3.2.2 Substrate Preference of *R. iodosum*

*R. iodosum* , a marine purple non‐sulfur bacterium, could also use all 4 organic substances, although not completely that is, only 82% ± 16% of butyrate and 27% ± 6% of glucose was consumed at the end of the experiment (Table 2, Figure S2). Organics and Fe(II) were consumed simultaneously in all cases. Lowest Fe(II) oxidation percentage was found with Fe(II) plus lactate (74% ± 11%) and Fe(II) plus butyrate (79% ± 3%) with over 93% oxidized in the case of Fe(II) only, Fe(II) plus acetate and Fe(II) plus glucose. For this marine strain *R. iodosum* , Fe(II) oxidation rate with Fe(II) only was 0.14 ± 0.03 mM/d, for Fe(II) plus lactate (0.12 ± 0.03 mM/d) and Fe(II) plus glucose (0.12 ± 0.02 mM/d) and within their standard deviation (Figure 1). Significantly higher Fe(II) oxidation rates were achieved with Fe(II) plus acetate (0.29 ± 0.03 mM/d, *p* = 0.003) whereas a lower rate was observed with Fe(II) plus butyrate (0.08 ± 0.03 mM/d) (Table S7). All measured cell numbers were higher than the theoretical calculation stated except for Fe(II) plus glucose where 7.3 ± 2.3 × 10^7^ cells/mL were measured (Figure 2). In this case lower cell numbers could also be related to the fact that only 27% ± 6% of glucose was used. In the case of Fe(II) plus acetate (6.6 ± 0.4 × 10^7^ cells/mL) and Fe(II) plus lactate (8.2 ± 0.2 × 10^7^ cells/mL) cell numbers were 1.5‐times higher than the theoretical value. Even higher values were measured in the setup with Fe(II) plus butyrate (2 times, 1.2 ± 0.2 × 10^8^ cells/mL) and Fe(II) only (3.5 times, 4.3 ± 0.9 × 10^7^ cells/mL). For the setup with Fe(II) and H_2_ a preferred usage of Fe(II) before H_2_ could be observed. Fe(II) oxidation started after a short lag phase of 6 days and at the end 90% ± 10% was oxidized with a rate of 0.05 ± 0.09 mM/d (Table 2, Figure S6). H_2_ was only used up to 1% ± 1% and did not differentiate much from the abiotic control.

In summary, *R. iodosum* could use all substrates except glucose partially and minimal H_2_. For organics and Fe(II), simultaneous consumption occurred, whereas with H_2_ sequential consumption occurred with preferential Fe(II) oxidation. Higher cell numbers than predicted could be observed in all setups except Fe(II) plus glucose.

#### 3.2.3 Substrate Preference of *R. robiginosum*

*R. robiginosum* , a marine purple non‐sulfur strain, could only partially use acetate (38% ± 9%) from the tested organic substrates and did so simultaneously with Fe(II) oxidation (Table 2, Figure S3). Fe(II) oxidation was also only partially completed (between 50% and 71%). Fe(II) oxidation rates were low with 0.027 ± 0.004 mM/d and the presence of lactate, butyrate or glucose appeared to have no impact. In case of Fe(II) plus acetate, Fe(II) oxidation was a little slower (0.018 ± 0.003 mM/d) than with Fe(II) only (Figure 1). Low cell numbers were also observed in the Fe(II) only setup with 4.9 ± 1.4 × 10^6^ cells/mL and all other setups were similar (Figure 2). In the experiments with Fe(II) and H_2_, 49% ± 10% of Fe(II) was oxidized with a Fe(II) oxidation rate of 0.06 ± 0.05 mM/d and 6% ± 7% of H_2_ used (Table 2, Figure S6). Although abiotic and biotic H_2_ data fluctuated and no trend in the biotic setup was observed. Thus, no clear conclusion concerning the consumption scenario of these substrates could be made.

In summary, *R. robiginosum* could only partially oxidize Fe(II), acetate, and H_2_ and had low Fe(II) oxidation rates and cell numbers across all conditions.

#### 3.2.4 Substrate Preference of *C. ferrooxidans* KoFox

In the organic and Fe(II) substrate experiment *C. ferrooxidans* KoFox could only use acetate simultaneously with Fe(II). Fe(II) oxidation extent was between 84% and 93% for all setups (Table 2, Figure S4). All Fe(II) oxidation rates were within the standard deviation of the Fe(II) only setup (0.27 ± 0.07 mM/d) (Figure 1). The lowest Fe(II) oxidation rates were with Fe(II) plus lactate at 0.17 ± 0.06 mM/d. For the freshwater strain *C. ferrooxidans* KoFox, cell numbers were lower than the theoretical values for Fe(II) only (5.0 ± 0.9 × 10^6^ cells/mL) (Figure 2). Cell numbers for Fe(II) plus acetate (1.4 ± 0.5 × 10^7^ cells/mL), Fe(II) plus lactate (1.3 ± 0.3 × 10^7^ cells/mL), and Fe(II) plus glucose (1.7 ± 0.6 × 10^7^ cells/mL) were within the range of the theoretical cell numbers for Fe(II) only and below the theoretical cell number for conditions with Fe(II) plus organics. In the experiment with Fe(II) and H_2_, simultaneous consumption was observed and both were consumed without a lag phase (Table 2, Figure S6). 90% ± 1% of Fe(II) was oxidized with a Fe(II) oxidation rate of 0.33 ± 0.36 mM/d and 40% ± 12% of H_2_ was used up. Both abiotic controls were stable throughout the experiment.

*C. ferrooxidans* KoFox could only utilize Fe(II), acetate, and H_2_, and both acetate and H_2_ were consumed simultaneously with Fe(II). Low cell numbers in the Fe(II) only setup were measured.

#### 3.2.5 Substrate Preference of *Chlorobium* sp. N1

*Chlorobium* sp. N1 could use acetate, lactate and glucose in the substrate preference experiment, but not butyrate. Fe(II) was 100% oxidized and all organics and Fe(II) were used simultaneously (Table 2, Figure S5). All Fe(II) oxidation rates with the organics present were higher than the rate of Fe(II) only (0.15 ± 0.01 mM/d) (Figure 1) although the Fe(II) oxidation rates in Fe(II) plus butyrate (0.18 ± 0.03) are not significantly higher than Fe(II) only. Rates for Fe(II) plus acetate and Fe(II) plus glucose are very similar (0.19 ± 0.01 and 0.18 ± 0.01 mM/d, respectively) and Fe(II) plus lactate rates increased to 0.38 ± 0.01 mM/d. For Fe(II) oxidation rates of Fe(II) plus acetate (*p* = 0.001), Fe(II) plus lactate (*p* < 0.001) and Fe(II) plus glucose (*p* = 0.008) changes were significant (Table S7). Although *Chlorobium* sp. N1 could use acetate, lactate and glucose, the cell numbers are only slightly increased for Fe(II) plus acetate (2.8 ± 1.0 × 10^7^ cells/mL), Fe(II) plus lactate (2.0 ± 0.6 × 10^7^ cells/mL), and Fe(II) plus glucose (2.2 ± 1.0 × 10^7^ cells/mL) compared to Fe(II) only (Figure 2). The cell numbers obtained for setups with Fe(II) plus butyrate (0.9 ± 0.3 × 10^6^ cells/mL) and Fe(II) only (1.1 ± 0.2 × 10^7^ cells/mL) were similar. Regarding the substrate preference between Fe(II) and H_2_, Fe(II) was oxidized before H_2_ was consumed (Table 2, Figure S6). Fe(II) was oxidized to 73% ± 6% with a Fe(II) oxidation rate of 0.09 ± 0.09 mM/d and only 3% ± 4% of H_2_ was consumed. Although growth on H_2_ was not observed during the experiment, growth with H_2_ was eventually observed later with *Chlorobium* sp. N1 after a lag phase of 30 days (Figure S7).

*Chlorobium* sp. N1 could use all substrates except glucose and Fe(II) oxidation rates were significantly increased. All organic compounds were used simultaneously with Fe(II), whereas Fe(II) was preferred in the case with H_2_.

#### 3.2.6 Substrate Preference of *R. palustris* TIE‐1

*R. palustris* TIE‐1, a freshwater purple non‐sulfur bacterium, could use all 4 organic substances (acetate, lactate, butyrate and glucose) completely. Acetate and butyrate were consumed first before Fe(II) oxidation proceeded whereas, with lactate and glucose simultaneous consumption with Fe(II) oxidation was observed in the substrate preference experiment (Table 2). Fe(II) was oxidized between 84% ± 5% (for Fe(II) plus acetate) and 98% ± 2% (for Fe(II) plus glucose). Fe(II) oxidation was significantly faster when both substrates were used simultaneously that is, the Fe(II) oxidation rates was 0.23 ± 0.04 mM/d with Fe(II) only and 1.1 ± 0.01 with lactate (*p* < 0.005) and 0.33 ± 0.04 mM/d with glucose (*p* = 0.02) (Figure 1). Fe(II) oxidation rate was slower with acetate (0.17 ± 0.01 mM/d) and within the standard deviation with butyrate (0.24 ± 0.01 mM/d). Cell numbers for *R. palustris* TIE‐1 were close to theoretical cell numbers except for the setup of Fe(II) plus glucose (Figure 2). Results for *R. palustris* TIE‐1 have also been described in detail in Nikeleit, Maisch, et al. (2024). For the substrate preference test with Fe(II) and H_2_ simultaneous consumption was observed (Table 2, Figure S6). No lag phase in both Fe(II) and H_2_ consumption was observed. The abiotic controls stayed stable except for 1 (H_2_) and 2 (Fe) timepoints. By the end 37% ± 5% of H_2_ and 64% ± 23% Fe(II) was oxidized with a Fe(II) oxidation rate of 0.18 ± 0.07 mM/d.

*R. palustris* TIE‐1 used all organics, Fe(II) and H_2_ showed sequential (for acetate and butyrate) and simultaneous (lactate, glucose, and H_2_) consumption, and Fe(II) oxidation rates were significantly increased with lactate and glucose.

## 4 Discussion

### 4.1 Influence of Co‐Substrates on Fe(II) Oxidation

One of the main objectives of this study was to determine whether photoferrotrophs continue to utilize Fe(II) when alternative electron donors are available. Overall, our results demonstrate that all investigated photoferrotrophs oxidized Fe(II) under photomixotrophic conditions and that Fe(II) oxidation occurred alongside the consumption of organic substrates or H_2_. Most tested photoferrotrophs oxidized all Fe(II) (> 80%), whilst *R. robiginosum* showed lowest Fe(II) oxidation extent (< 80%), Fe(II) oxidation rates and cell numbers.

Our tested photoferrotrophs show high metabolic flexibility and were able to use Fe(II) with other organic and/or inorganic substrates. Generally, Fe(II) was oxidized simultaneously with the other co‐substrates, although it is still unclear whether it is possible for one cell to simultaneously metabolize multiple substrates or whether the simultaneous consumption arises from community effects where parts of the population specialize in one specific substrate.

In the case of H_2_ consumption, our data show a trend of simultaneous consumption with freshwater strains and sequential consumption with marine strains. The observed preference for Fe(II) may reflect an environmental effect rather than an enzyme‐mediated species‐specific response. Hydrogen solubility decreases with increasing salinity, potentially reducing its availability as an electron donor in marine media. Based on calculations by Zhu et al. (2022) hydrogen solubility is reduced by ~13.7% at 2.7% salinity (*Chlorobium* sp. N1 media) and by 20% at 4% salinity (*R. robiginosum and R. iodosum* media) relative to freshwater conditions. This reduced hydrogen availability may have favoured the utilization of Fe(II) as an alternative electron donor.

Other exceptions were observed with *R. palustris* TIE‐1 and discussed by Melton et al. (2014) and Nikeleit, Maisch, et al. (2024). Ehrenreich and Widdel (1994) also found that *R. ferrooxidans* SW2 sequentially used acetate or glucose before oxidizing Fe(II). However, their study used higher concentrations in general and a different Fe(II) source (FeSO_4_; 1 to 10 mM). The use of higher Fe(II) concentrations can also cause longer lag phases and therefore potentially influence the order of consumption. *R. ferrooxidans* SW2 grew in this study with 2 mM Fe(II) and had no lag phase whereas with higher Fe(II) concentrations of 10 mM lag phases of 5 and 12 days have been reported (Ehrenreich and Widdel 1994; Nikeleit, Mellage, et al. 2024). This suggests that substrate preferences are at least partly concentration dependent. As we used environmentally relevant substrate concentrations, the simultaneous substrate consumption observed in this study is likely more representative of natural environments.

### 4.2 Influence of Co‐Substrates on Fe(II) Oxidation Rates

Although it might be expected that the presence of organic substrates would hinder Fe(II) oxidation, we observe that several strains oxidize Fe(II) significantly faster in the presence of specific co‐substrates. Higher Fe(II) oxidation rates were observed with the organic co‐substrate acetate (*R. ferrooxidans* SW2, *R. iodosum* and *Chlorobium* sp. N1), lactate ( *R. palustris* TIE‐1 and *Chlorobium* sp. N1), glucose ( *R. palustris* TIE‐1 and *Chlorobium* sp. N1) and pyruvate ( *R. palustris* TIE‐1; Nikeleit, Maisch, et al. 2024). In all other setups, Fe(II) oxidation rates were unaffected or mildly reduced by the presence of organics. Importantly, we did not observe any condition where Fe(II) oxidation was inhibited.

This observation is consistent with earlier studies; for example, Ehrenreich and Widdel (1994) observed an increase in Fe(II) oxidation of *R. ferrooxidans* SW2 with acetate and glucose. In their case, due to sequential consumption with preference for the organics, they attributed this Fe(II) oxidation rate increase to higher cell numbers. In our study, we could confirm the increase of Fe(II) oxidation rate of *R. ferrooxidans* SW2 at lower concentrations with acetate during simultaneous consumption, but not with glucose. Similarly, for the first isolated green sulfur photoferrotroph *C. ferrooxidans* KoFox, Heising et al. (1999) also observed accelerated Fe(II) oxidation with acetate, pyruvate, fumarate, cysteine, and thiosulfate. More cells of *C. ferrooxidans* KoFox were observed with acetate, pyruvate, and fumarate, whereas in the case of pyruvate and fumarate, an increase of the partner strain *Geospirillum* KoFum was also observed. Although in these cases our study generally confirms previous observations, earlier experiments were conducted with higher concentrations which are not environmentally relevant.

Since our study also included all 3 isolated marine strains, we could evaluate whether the presence of organic substrates could be influential for marine photoferrotrophs. For *Chlorobium* sp. N1, the addition of organics had a large positive impact on Fe(II) oxidation rates. Whereas for *R. robiginosum* no significant increase and for *R. iodosum* only a significant increase of Fe(II) oxidation rates with acetate was observed. The elevated Fe(II) oxidation rates observed with acetate may be related to the need for additional reducing equivalents. Acetate is more oxidized than butyrate which can provide more reducing power during metabolism. As a result, cells growing on acetate may benefit more from electrons derived from Fe(II) oxidation. Supporting this idea, Haas et al. (2022) showed that *Rhodopseudomonas palustris* CGA010 oxidized Fe(II) during photoheterotrophic growth on the relatively oxidized carbon substrate malate. In that study, Fe(II) oxidation supplied additional reducing equivalents and increased growth yield, and expression of the pioABC genes was linked to the oxidation state of the carbon substrate.

In contrast to the relatively positive impact of organics on Fe(II) oxidation rates, we observed that Fe(II) oxidation rates were generally lower in the presence of H_2_. The same trend was also observed by Croal et al. (2009) where the Fe(II) oxidation rate was reduced for *R. palustris* TIE‐1 (from 0.13 to 0.09 mM/h) and *R. ferrooxidans* SW2 (from 0.46 to 0.28 mM/h) in cell suspension experiments. In contrast, Ehrenreich and Widdel (1994) reported a positive effect of H_2_ on Fe(II) oxidation by *R. ferrooxidans* SW2, and Heising et al. (1999) observed a similar stimulatory effect for *C. ferrooxidans* KoFox. This contrasts with our observation, but we also used a different Fe(II) source, lighting, and lower Fe(II) concentration which can explain these discrepancies. Lower Fe(II) oxidation rates when grown with H_2_ may be related to the fact similar proteins are required to metabolize both substrates which could lead to competition (Bryce, Franz‐Wachtel, et al. 2018). In the case of *C. ferrooxidans* KoFox and *R. robiginosum* , Fe(II) oxidation rates with H_2_ were increased but showed large variation.

In summary, organic co‐substrates such as acetate, lactate, and glucose enhanced Fe(II) oxidation rates in several photoferrotrophs, though the effect varied by strain and substrate. We could not detect a specific trend for freshwater compared to marine strains. Hydrogen (H_2_) generally reduced Fe(II) oxidation rates, although some strains showed increased rates.

### 4.3 Influence of Co‐Substrates on Growth

In general, the observed cell abundances broadly reflected both the extent of Fe(II) oxidation and the theoretically calculated utilization of the additional organic substrates. The calculations were based on the framework of McKinlay and Harwood (2010), assuming that ~80% of acetate is converted into biomass while the remaining fraction is oxidized to provide reducing power and energy for growth. Under these assumptions, the predicted yields corresponded well with the observed cell numbers of *R. palustris* TIE‐1 and *R. ferrooxidans* SW2. However, *R. iodosum* consistently achieved higher cell abundances than predicted, indicating a potentially greater efficiency of substrate assimilation or additional sources of electrons and carbon that were not considered in the model. The low cell abundances of *R. robiginosum* corresponded with its comparatively low Fe(II) oxidation extent, as not all Fe(II) was oxidized and acetate was only partially consumed.

A notable exception was observed for the green sulfur bacteria. Despite differences in substrate utilization and Fe(II) oxidation rates between treatments, cell abundances remained relatively similar across conditions. This indicates that additional substrate consumption did not necessarily translate into proportional increases in biomass. One explanation is that the investigated organisms differ in how efficiently organic substrates are converted into biomass and how electrons derived from Fe(II) oxidation are partitioned between growth, cell maintenance, and carbon fixation.

Green sulfur bacteria are fundamentally photolithoautotrophic organisms whose growth is largely supported by light‐driven electron transport and CO_2_ fixation via the reverse tricarboxylic acid (rTCA) cycle, such that assimilated organic compounds typically supplement rather than replace autotrophic carbon assimilation (Lambrecht et al. 2021; Kushkevych et al. 2024; Feng et al. 2010).

Metabolic flux analyses of the green sulfur bacterium *Chlorobaculum tepidum* showed that even during mixotrophic growth on acetate or pyruvate, most biomass carbon continued to originate from CO_2_ fixation through the rTCA cycle, while organic substrates primarily supplemented central carbon metabolism. The same study reported a trade‐off between carbon fixation and growth, indicating that increased substrate assimilation and carbon incorporation do not necessarily result in proportional increases in cell abundance (Feng et al. 2010).

Purple phototrophs, in contrast, may incorporate organic substrates more efficiently into central metabolism during photomixotrophic growth and are generally characterized by high biomass yields on organic substrates owing to their considerable metabolic versatility (Cerruti et al. 2022; Kushkevych et al. 2021). They can utilize a wide range of organic compounds as carbon and electron sources while simultaneously harvesting light energy, allowing organic carbon to be incorporated directly into central metabolic pathways and biomass production rather than relying predominantly on CO_2_ fixation. As a result, substrate consumption is often more closely linked to cell growth than in green sulfur bacteria, which remain strongly dependent on autotrophic carbon fixation even during mixotrophic growth.

While both green sulfur bacteria and purple phototrophs are capable of autotrophic growth, purple phototrophs may incorporate organic substrates more efficiently into central metabolism during photomixotrophic growth. Consequently, a larger fraction of electrons derived from Fe(II) oxidation in *Chlorobium* may be directed towards maintenance metabolism and carbon fixation rather than cell division.

### 4.4 Inferred Genetic Potential Compared to Actual Potential

Understanding the interplay between genetic potential and actual activity in bacteria is crucial for unravelling their adaptive strategies, evolutionary processes, and interactions with their surroundings (Kobras et al. 2021). In several cases, discrepancies between the predicted substrate usage in the genomes, previously reported metabolic capabilities, and the substrate use in this study (Table 3). Generally, we observed that substrate usage was consistent with that observed in the original descriptions of the strains and their metabolic capabilities. However, several notable exceptions were identified. For example, where Heising et al. (1999) observed growth of *R. robiginosum* with lactate, which was not observed in this study. The long‐time span between the original isolation studies and the present work may also contribute to some of the observed differences. More than 20 years have passed between our study and many of the isolation and characterization papers, with many of the strains maintained in continuous culture. In extreme cases, bacteria can lose the ability to use certain substrates due to mutation or loss of the genes (Albalat and Cañestro 2016).

**TABLE 3:** Comparison of genetic and actual potential of 5 different photoferrotrophs.

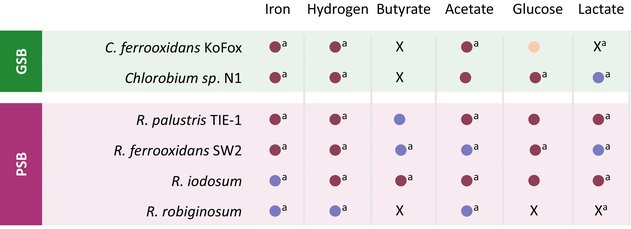

*Note:* Beige circles (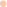) indicate genetic potential, violet circles (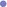) indicate observed consumption and dark red circles (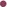) indicate genetic and observed consumption in this study.

^a^ Indicates that growth has been shown in previous publications (Ehrenreich and Widdel 1994; Heising et al. 1999; Jiao et al. 2005; Laufer et al. 2017; Straub et al. 1999). For R. robiginosum no genome is available.

Conversely, in the case of *R. palustris* TIE‐1 we showed for the first time that growth with butyrate and glucose was possible. Similarly, *R. iodosum* also grew with glucose, which was not previously observed.

Differences were also observed between genomic potential substrate consumption and what we observed in this study. Four photoferrotrophs exhibited at least one difference, resulting in a total of 8 mismatched substrates. Despite our careful selection of marker genes, false negative results may be explained by alternative substrate utilization pathways that have yet to be characterized or by lack of completeness of the sequenced genomes, although this is less likely due to high estimates of genome completeness in all cases. For *R. iodosum* and *R. robiginosum*, genes for Fe(II) oxidation have not been identified yet but both have shown clear evidence for Fe(II) oxidation. In the case of both *Chlorobium* strains, it is also known that they have a partner strain which comprises < 1% of the whole community but which may contribute to the overall ability of the culture to utilize certain substrates (Bryce et al. 2019; Heising et al. 1999). Our analysis of the *C. ferrooxidans* KoFox genome shows that glucose consumption could be possible but was not observed in our and previous studies (Heising et al. 1999).

Overall, these results highlight an important limitation of genome‐based functional predictions. Particularly in the age of metagenomics, functional predictions are still predictions, rather than direct evidence of activity. There are usually many steps involved in the utilization of a given substrate beyond the transcription of a distinctive marker gene. Cultivation‐based studies remain essential in determining the genuine metabolic capabilities of bacteria.

### 4.5 Ecological Implications

Metabolic flexibility can enhance the ecological competitiveness of photoferrotrophs against other bacteria. Many other phototrophs and heterotrophs can also use organics as an electron and carbon source and it is estimated that chemoorganotrophs grow faster than photolithotrophs (Raven et al. 2013). Further, being able to oxidize Fe(II) is an exotic trait for bacteria and phototrophs and only a small percentage have been identified and studied. There are only two other categorized Fe(II)‐oxidizers at neutral pH which are in direct competition for Fe(II): Microaerophilic and nitrate‐dependent Fe(II)‐oxidizers. Although they are competing for Fe(II), these microbes have been found active simultaneously in the same environments (Laufer et al. 2016; Otte et al. 2018). Additionally, iron is needed by all bacteria in small concentrations for necessary proteins like cytochromes, nitrogenase, catalase, and peroxidase but in higher concentrations it can be a stressor, growth inhibiting, and even toxic for some bacteria (Ernst et al. 2022; Ratledge and Winder 1964; Sorokina et al. 2013).

With the ability to use Fe(II) as an additional electron source, photoferrotrophs can expand their ecological niche and have been found in a variety of environments like sediments (freshwater and marine), stratified lakes and soils (Ehrenreich and Widdel 1994; Lambrecht et al. 2021; Llirós et al. 2015; Otte et al. 2018). They also thrive in iron‐rich ponds and springs and can cope with high iron concentrations through iron homeostasis (Bryce, Franz‐Wachtel, et al. 2018; Jiao et al. 2005). The ability to respond to fluctuations in nutrient availability by enabling efficient utilization of different energy sources is an extremely useful trait in the environment. This is particularly important for phototrophs in marine environments, where nutrient levels can vary widely, some daily or in dimictic stratified lakes with seasonal changes (Hojerová et al. 2011; Swanner et al. 2022). Photoferrotrophs coexist with a range of potential competitors and are exposed to different organic and inorganic electron donors. The ability to use Fe(II) next to other substrates likely contributes to their persistence in freshwater and marine sediments, ferruginous water columns, and iron‐rich springs (Bryce, Blackwell, et al. 2018; Crowe et al. 2017; Laufer et al. 2017; Otte et al. 2018; Walter et al. 2014). Furthermore, because Fe(II) oxidation drives Fe(III) mineral formation, continued Fe(II) oxidation in the presence of alternative substrates suggests that photoferrotrophs may exert a larger influence on mineral formation and nutrient cycling than would be expected if substrate competition strongly suppressed photoferrotrophy. This capacity for photomixotrophic growth and sustained Fe(II) oxidation supports their importance in modern ferruginous ecosystems.

## 5 Conclusion

The ability of photoferrotrophs to use multiple substrates is an advantage for these microorganisms. We determined that Fe(II) oxidation rates are enhanced or, at worst, unaffected by organic substrates. Generally, most substrates were used simultaneously with Fe(II). Thus, in contrast to the expectation that the presence of organic co‐substrates in the environment would hinder Fe(II) oxidation in phototrophs, it is the case that co‐substrate usage can instead improve the fitness of these photoferrotrophs and enhance their ecological competitiveness. Contrastingly, H_2_ utilization, which requires much of the same cellular machinery as Fe(II) oxidation, can lead to a decrease in the extent of Fe(II) oxidation. The present study demonstrates that photoferrotrophs are likely to be conducting Fe(II) oxidation when the environment is favourable, even when other substrate options are available. This has significance for the ubiquity and importance of photoferrotrophy in the natural environment.

## Author Contributions

**Verena Nikeleit:** conceptualization, investigation, writing – original draft, visualization, validation, methodology, data curation, formal analysis. **Giorgio Bianchini:** methodology, data curation, writing – review and editing, visualization, validation, formal analysis. **Jimena C. Lopez‐Rivoldi:** data curation, writing – review and editing. **Marie Mergenthaler:** data curation, writing – review and editing. **Josia Reinhold:** data curation, writing – review and editing. **Patricia Sanchez‐Baracaldo:** supervision, writing – review and editing, resources. **James M. Byrne:** formal analysis, writing – review and editing, funding acquisition, project administration, conceptualization. **Andreas Kappler:** resources, supervision, project administration, funding acquisition, conceptualization, writing – review and editing, methodology. **Casey Bryce:** funding acquisition, conceptualization, project administration, supervision, resources, writing – original draft, methodology, validation, investigation.

## Funding

This work was supported by Deutsche Forschungsgemeinschaft, BR 5927/2‐1, BY 82/4‐1. UKRI Future Leaders Fellowship UKRI2857.

## Conflicts of Interest

The authors declare no conflicts of interest.

## Supporting information


**Figure S1:** Growth of R. ferrooxidans SW2 with different growth substrates. R. ferrooxidans SW2 was grown with Fe(II) plus acetate (A), lactate (B), butyrate (C), glucose (D) and with Fe(II) alone (E). The standard deviation is based on biological triplicates.
**Figure S2:** Growth of *R. iodosum* with different growth substrates. *R. iodosum* was grown with Fe(II) plus acetate (A), lactate (B), butyrate (C), glucose (D) and with Fe(II) alone (E). The standard deviation is based on biological triplicates.
**Figure S3:** Growth of *R. robiginosum* with different growth substrates. *R. robiginosum* was grown with Fe(II) plus acetate (A), lactate (B), butyrate (C), glucose (D) and with Fe(II) alone (E). The standard deviation is based on biological triplicates.
**Figure S4:** Growth of *C. ferrooxidans* KoFox with different growth substrates. *C. ferrooxidans* KoFox was grown with Fe(II) plus acetate (A), lactate (B), butyrate (C), glucose (D) and with Fe(II) alone (E). The standard deviation is based on biological triplicates.
**Figure S5:** Growth of Chlorobium sp. N1 with different growth substrates. Chlorobium sp. N1 was grown with Fe(II) plus acetate (A), lactate (B), butyrate (C), glucose (D) and with Fe(II) alone (E). The standard deviation is based on biological triplicates.
**Figure S6:** Panels show the substrate preference of *R. palustris* TIE‐1, R. ferrooxidans SW2, *R. iodosum* , *R. robiginosum* , *C. ferrooxidans* KoFox and Chlorobium sp. N1 incubated with Fe(II) and H_2_. Biotic data are from triplicates from sacrificial setup and abiotic sample show data from one bottle.
**Figure S7:** Growth of Chlorobium sp. N1 on H_2_ after 30 days.
**Table S1:** Media composition for each photoferrotroph.
**Table S7:** T‐test with Fe(II) oxidation rates between Fe(II) only and Fe(II) oxidation rates with organics.

**Table S2:** Accession numbers for genome assemblies analysed in this study.
**Table S3:** Accession numbers and GA thresholds for HMMs used in this study. For entries retrieved from the NCBI Protein Models database, the GA thresholds were extracted from the HMM files; for HMMs gathered from FeGenie, thresholds were extracted from https://github.com/Arkadiy‐Garber/FeGenie/blob/master/hmms/iron/HMM‐bitcutoffs.txt.
**Table S4:** BLAST query accessions for sequences retrieved from UniProt and from previous studies. For each gene, a curated set of homologues (“ingroup”) was retrieved from the specified source, and 10 representative query sequences were selected. To determine the score threshold, the query sequences for each gene were BLASTed against the corresponding ingroup. For each ingroup sequence, the highest observed bit score was identified, and the score threshold was determined as the minimum of these scores. Following this procedure, we expect “true” hits within the genomes we analysed to obtain a score higher than the threshold, for at least one query sequence; this was treated as a “soft” threshold, and hits close to this value were manually inspected to confirm their identity.
**Table S5:** BLAST results. In the top part, for each gene and proteome, the table shows all BLASTp hits with scores above the threshold (black). For proteomes where no such hit was found, the highest‐scoring hit is shown in light grey. For each hit, the accession number of the protein product, as well as the bit score and the e‐value are shown. In the bottom part, the table shows the highest‐scoring tBLASTn hits for genomes where each gene was not found within the annotated proteome. In the case of C. ferrooxidans DSM 13031, this revealed a pscA homologue that was not annotated in the proteome (highlighted in red). In all other cases, the hits are shown in light grey.
**Table S6:** HMMer and BATH results. In the top part, for each gene and proteome, the table shows all HMMer hits with scores above the GA threshold (black). For proteomes where no such hit was found, the highest‐scoring hit is shown in light grey. For each hit, the accession number of the protein product, as well as the score and the e‐value are shown. In the bottom part, the table shows the highest‐scoring BATH hits for genomes where each gene was not found within the annotated proteome. These are all shown in light grey, as no additional homologues were found.

## Data Availability

The data that supports the findings of this study are available in the supplements of this manuscript.
