# Supplementary material for: Phototrophic Fe(II)‐Oxidizers Can Use Multiple (In)Organic Substrates Simultaneously
Source: Environ Microbiol Rep. 2026 Nov 4;18(6):e70428. doi: 10.1111/1758-2229.70428 (PMC13732420; doi:10.1111/1758-2229.70428)
Supplement: Supplementary file 1 [file EMI4-18-e70428-s002.docx]

**Supplements**

**Phototrophic Fe(II)-oxidizers can use multiple (in)organic substrates simultaneously**

Verena Nikeleit^1,2*^, Giorgio Bianchini^3,4^, Jimena C. Lopez-Rivoldi^1^, Marie Mergenthaler^1^, Josia Reinhold^1^, Patricia Sanchez-Baracaldo^3^, James M. Byrne^5^, Andreas Kappler^1,6^ and Casey Bryce^6*^

^1^Department of Geoscience, University of Tübingen, Tübingen, Germany

^2^NOW: NORCE Norwegian Research Center AS, 5008 Bergen, Norway

^3^School of Geographical Sciences, University of Bristol, Bristol, UK

^4^NOW: Department of Life Sciences, University of Bath, Bath, UK

^5^School of Earth Sciences, University of Bristol, Bristol, UK

^6^Cluster of Excellence: EXC 2124: Controlling Microbes to Fight Infections, Tübingen, Germany

*Corresponding Authors:

1) Casey Bryce, School of Earth Science, Wills Memorial Building, University of Bristol, Queens Road, Bristol, BS8 1RJ, UK; Email: [casey.bryce@bristol.ac.uk](mailto:casey.bryce@bristol.ac.uk)

For submission to Environmental Microbiology

KEYWORDS: photoferrotroph, metabolic flexibility, Fe(II) oxidation

*Table S1: Media composition for each photoferrotroph.*

| Organism | Salts | Buffer | Additives |
| --- | --- | --- | --- |
| *R. iodosum* and  *R. robiginosum* | 26.4 g/L NaCl  5.7 g/L MgSO_4_·7 H_2_O,  5.7 g/L MgCl_2_·6 H_2_O, 1.5 g/L CaCl_2_·2 H_2_O,  0.66 g/L KCl,  0.09 g/L Br  0.6 g/L KH_2_PO_4_ and  0.3 g/L NH_4_Cl were autoclaved separately | 2.5 gL/ NaHCO_3_ buffer | 1 mL/L sterile filtered 7-vitamin solution (Widdel & Pfenning, 1981),  1 mL/ trace element solution (Widdel, 1983) and  1 m/L selenite-tungstate solution (Widdel & Bak, 1992)  0.5 mL/L Na_2_S_2_O_3_ (1 M) |
| *C. ferrooxidans* KoFox | 0.0025 g/L MgSO_4_·7 H_2_O,  0.4 g/L MgCl_2_·6 H_2_O, 0.1 g/L CaCl_2_·2 H_2_O  0.6 g/L KH_2_PO_4_,  0.3 g/L NH_4_Cl | 1.85 gL/ NaHCO_3_ buffer | 1 mL/L sterile filtered 7-vitamin solution (Widdel & Pfenning, 1981),  1 mL/ trace element solution (Widdel, 1983) and  1 m/L selenite-tungstate solution (Widdel & Bak, 1992) |
| *R. palustris* TIE-1 | 0.5 g KH_2_PO_4_,  0.3 g NH_4_Cl,  0.5 g MgSO_4_ * 7H_2_O, 0.1 g CaCl_2_*2H_2_O | 1.85 gL/ NaHCO_3_ buffer | 10 mL/L filter-sterilized vitamin solution (altered from Ehrenreich & Widdel, 1994; containing 50 mg/L riboflavin), 1 mL/L SL10 trace element solution (Widdel, 1983), 1 mL/L selenite-tungstate solution (Widdel & Bak, 1992) and 0.1mg/L vitamin B12 solution (c=100 mg/L) were added |
| *Chlorobium* sp. N1 | 17.3 g/L NaCl  0.025 g/L MgSO_4_·7 H_2_O,  8.6 g/L MgCl_2_·6 H_2_O, 0.99 g/L CaCl_2_·2 H_2_O,  0.39 g/L KCl,  0.059 g/L Br  0.05 g/L KH_2_PO_4_ and  0.25 g/L NH_4_Cl were autoclaved separately | 1.85 gL/ NaHCO_3_ buffer | 1 mL/L sterile filtered 7-vitamin solution (Widdel & Pfenning, 1981),  1 mL/ trace element solution (Widdel, 1983) and  1 m/L selenite-tungstate solution (Widdel & Bak, 1992) |
| *R. ferrooxidans* SW2 | 0.6 g/L KH_2_PO_4_,  0.3 g/L NH_4_Cl,  0.025 g/L MgSO_4_·7 H_2_O,  0.4 g/L MgCl_2_·6 H_2_O, 0.1 g/L CaCl_2_·2 H_2_O | 1.85 gL/ NaHCO_3_ buffer | 1 mL/L sterile filtered 7-vitamin solution (Widdel & Pfenning, 1981),  1 mL/ trace element solution (Widdel, 1983) and  1 m/L selenite-tungstate solution (Widdel & Bak, 1992) |

*Table S7: T-test with Fe(II) oxidation rates between Fe(II) only and Fe(II) oxidation rates with organics.*

| ***R. iodosum*** | | **p-value** |
| --- | --- | --- |
| Fe only | Fe plus Ac | 0.0026 |
|  | Fe plus Lc | 0.3644 |
|  | Fe plus Bu | 0.0582 |
|  | Fe plus Glu | 0.4509 |
|  | | |
| ***C.ferrooxidans* KoFox** | | **p-value** |
| Fe only | Fe plus Ac | 0.4827 |
|  | Fe plus Lc | 0.6227 |
|  | Fe plus Bu | 0.2057 |
|  | Fe plus Glu | 0.0767 |
|  | | |
| ***Chlorobium* N1** | | **p-value** |
| Fe only | Fe plus Ac | 0.0012 |
|  | Fe plus Lc | 8E-07 |
|  | Fe plus Bu | 0.1174 |
|  | Fe plus Glu | 0.008 |
|  | | |
| ***R. robiginosum*** | | **p-value** |
| Fe only | Fe plus Ac | 0.0515 |
|  | Fe plus Lc | 0.1398 |
|  | Fe plus Bu | 0.2064 |
|  | Fe plus Glu | 0.9604 |
|  | | |
| ***R. ferrooxidans* SW2** | | **p-value** |
| Fe only | Fe plus Ac | 0.0053 |
|  | Fe plus Lc | 0.2885 |
|  | Fe plus Bu | 0.4915 |
|  | Fe plus Glu | 0.8195 |

Figure S1: Growth of *R. ferrooxidans* SW2 with different growth substrates. *R. ferrooxidans* SW2 was grown with Fe(II) plus acetate (A), lactate (B), butyrate (C), glucose (D) and with Fe(II) alone (E). The standard deviation is based on biological triplicates.

Figure S2: Growth of *R. iodosum* with different growth substrates. *R. iodosum* was grown with Fe(II) plus acetate (A), lactate (B), butyrate (C), glucose (D) and with Fe(II) alone (E). The standard deviation is based on biological triplicates.

Figure S3: Growth of *R. robiginosum* with different growth substrates. *R. robiginosum* was grown with Fe(II) plus acetate (A), lactate (B), butyrate (C), glucose (D) and with Fe(II) alone (E). The standard deviation is based on biological triplicates.

Figure S4: Growth of *C. ferrooxidans* KoFox with different growth substrates. *C. ferrooxidans* KoFox was grown with Fe(II) plus acetate (A), lactate (B), butyrate (C), glucose (D) and with Fe(II) alone (E). The standard deviation is based on biological triplicates.

Figure S5: Growth of *Chlorobium sp*. N1 with different growth substrates. *Chlorobium sp.* N1 was grown with Fe(II) plus acetate (A), lactate (B), butyrate (C), glucose (D) and with Fe(II) alone (E). The standard deviation is based on biological triplicates.

*Figure S6: Panels show the substrate preference of R. palustris* TIE-1*, R. ferrooxidans* SW2*, R. iodosum, R. robiginosum, C. ferrooxidans* KoFox *and Chlorobium sp.* N*1 incubated with Fe(II) and H_2_. Biotic data are from triplicates from sacrificial setup and abiotic sample show data from one bottle.*

*Figure S7: Growth of Chlorobium sp.* N1 *on H_2_ after 30 days.*
